# Reporting guidelines: doing better for readers

**DOI:** 10.1186/s12916-018-1226-0

**Published:** 2018-12-14

**Authors:** David Moher

**Affiliations:** 1 Centre for Journalology, Clinical Epidemiology Program, The Ottawa Hospital Research Institute, The Ottawa Hospital - General Campus, 501 Smyth Rd, Room L1288, Ottawa, ON K1H 8L6 Canada; 2 School of Epidemiology and Public Health, Faculty of Medicine, University of Ottawa, Ottawa, Canada

**Keywords:** Quality of reporting, EQUATOR, reporting guidelines, evidence, reproducibility, audit and feedback

## Abstract

There is clear guidance on the responsibilities of editors to ensure that the research they publish is of the highest possible quality. Poor reporting is unethical and directly impacts patient care. Reporting guidelines are a relatively recent development to help improve the accuracy, clarity, and transparency of biomedical publications. They have caught on, with hundreds of reporting guidelines now available. Some journals endorse reporting guidelines while a smaller number have used various approaches to implement them. Yet challenges remain – biomedical research is still not optimally reported despite the abundance of reporting guidelines. Electronic algorithms are now being developed to facilitate the choice of correct reporting guideline(s), while other tools are being integrated into journal editorial management processes. Universities need to consider whether it is responsible to advance careers of faculty based on poorly reported research which is of little societal value. If journals embraced auditing of the quality of articles they publish this would give them and their readers essential feedback from which to improve their product.

## Background

In 2003, the year when *BMC Medicine* was first launched, few reporting guidelines existed [1] despite ample evidence of poor reporting across biomedical research. In 1980, Altman [2] noted that the misuse of statistics, and their reporting, was unethical. Later, Rennie, then deputy editor at *JAMA*, observed “*there seems to be no study too fragmented, no hypothesis too trivial, no literature citation too biased or too egotistical, no design too warped, no methodology too bungled, no presentation of results too inaccurate, too obscure, and too contradictory, no analysis too self-serving, no argument too circular, no conclusions too trifling or too unjustified, and no grammar and syntax too offensive for a paper to end up in print*” [3]. Chalmers and Glasziou [4] reminded us of the substantial and avoidable fiscal waste associated with a plethora of problems of biomedical research, including inadequate reporting. Indeed, bad reporting directly impacts patient care [5, 6].

As *BMC Medicine* celebrates its 15th anniversary, it is useful to examine whether there are now mechanisms in place to improve the clarity, accuracy, and transparency of publications – a fundamental goal and responsibility of any credible journal [7].

### Evolution of better reporting

Using the CONSORT statement as a launching pad, the EQUATOR Network was established in 2006. The vision was to develop a broad basket of tools to help authors, editors, peer reviewers, and others improve the reporting of articles published in biomedicine. The network was formally launched in 2008 [8]. Today, the network is well on the way to meeting its initial remit, with the EQUATOR library being an open repository of more than 400 reporting guidelines developed or currently under development [9]. The network also developed guidance to help others interested in developing a reporting guideline [10], as well as several toolkits for multiple stakeholders, including guidance for authors writing manuscripts, manuscript peer reviewers, and editors wanting to implement reporting guidelines at their journal. All four EQUATOR centers (Australasia, Canada, France, and UK) have publication schools to help authors, particularly early career ones, produce better reports for publication consideration. The algorithm-based EQUATOR Wizard is an initial attempt to help prospective authors identify the most appropriate reporting guideline to use when reporting their research [11]. Other groups, such as the REWARD Alliance (http://rewardalliance.net/), are also drawing attention to these problems and offering solutions. Machine reading tools that provide automatic and immediate assessment of reporting guideline compliance, e.g., CONSORT in the first instance [12], are also starting to appear to help authors and editors. These schemes are now being integrated into editorial management systems and such developments could enable editors invoke quality compliance thresholds below which a manuscript cannot be formally submitted to a journal.

While authors sometimes submit shoddy reports for publication consideration, peer reviewers offer a potential screen of theoretically acceptable publications. Indeed, *BMC Medicine* has drawn attention to peer review and called for greater professionalism of it [13]. Journals could invoke their own quality threshold by insisting upon using only certified peer reviewers. Peer review supplemented with the use of reporting guidelines is likely to improve the peer-review report and quality of the manuscript under assessment, although more data is required to substantiate this claim. However, it is hard to imagine that using reporting guidelines would provide less informative peer-review reports.

It is possible that the misguided ‘publish or perish’ mantra at academic centers is promoting unscientific and unethical behavior when authors report their research. The prevalence rates of reporting biases are disturbingly high [14, 15], and why researchers would get promoted for such offences is difficult to understand. It is possible that more widespread uptake of declarations of transparency by journals would reduce these reporting biases in publications [16]. Similarly, Universities should consider modifying their incentive criteria towards rewarding career advancement based on better quality publications rather than on quantity. Such a policy directive might also contribute to improving the value of biomedical publications to society.

### Challenges

There are now plenty of reporting guidelines to help authors, editors, and peer reviewers, yet several challenges remain. Whether there is reporting guideline inflation resulting in potential confusion for users requires serious reflection. While there is accumulating evidence that use of reporting guidelines is associated with improved reporting, albeit not in all cases [17], this evidence base is limited only to a few reporting guidelines [18, 19]. Additionally, reporting guideline developers seem hesitant to provide this essential data, likely due to the considerable problems in funding such endeavors. However, such as with pharmaceuticals, we should be more cautious about recommending the use of reporting guidelines without evidence of effectiveness. Further, even armed with an initial evidence base about the effectiveness of reporting guidelines, few editors recommend their peer reviewers use them [20]; thus, we need to enhance all implementation efforts [21]. Finally, any attempt to investigate reproducibility is more likely to be possible when reports are clearly and accurately reported; otherwise, such attempts are difficult to initiate [22].

Readers have little information about the quality of what journals publish. Journals need to be more proactive in providing this information such as through regular audits and feedback. Auditing the quality of reports that a journal publishes might provide important information for authors, editors, peer reviewers, and readers in identifying problems and opportunities to enhance the quality of published articles. Making such information available to the public would send a strong positive signal about openness, sharing data, and the journal’s commitment to continuous quality improvement.

Funding programs to improve the quality of reporting biomedical research is remarkably difficult. The irony is that there is volumetric evidence of enormous avoidable waste in the current reporting of biomedical research and why there is hesitancy to fund programs to help improve the quality of reporting is difficult to comprehend. While a few enlightened funders are recognizing the importance of funding such research as well as other journalology and meta-research, there is much to be done to galvanize the majority of funders [23].

## Conclusions

Clear, accurate, and transparent reporting of biomedical research remains a considerable problem. Authors, editors, and peer reviewers have failed readers in providing a product that is robust, usable, and reproducible. There are now innovative tools available to help improve this situation, yet we need more active implementation of them by authors, editors, and peer reviewers. University promotion and tenure committees should consider whether offering career advancement based on poorly reported publications is of value to society and ethically sustainable.
